# Exploring the mechanism of dendrobine in treating metabolic associated fatty liver disease based on network pharmacology and experimental validation

**DOI:** 10.1186/s41065-024-00322-2

**Published:** 2024-05-16

**Authors:** Feng Li, Jialin Wu, Ye Zhu, Xiaoyan Zhang, Miao Wang, Shigao Zhou

**Affiliations:** 1 Traditional Chinese Medicine Department, Longhua Hospital, Shanghai University of Traditional Chinese Medicine, 725 Wanping South Road, Fenglin Street, Xuhui District, Shanghai, 200030 China; 2 Nanmatou Community Health Service Center, 696 Pusan Road, Pudong New District, Shanghai, 200125 China; 3 Xinzhuang Community Health Service Center, 115 Xinjian Road, Minhang District, Shanghai, 201199 China; 4 Shanghai University of Traditional Chinese Medicine, School of Traditional Chinese Medicine, Shanghai, 201203 China

**Keywords:** Metabolic associated fatty liver disease, Dendrobine, Network pharmacology, Experimental validation, Clinical trials

## Abstract

**Background:**

This study investigates the therapeutic mechanisms of dendrobine, a primary bioactive compound in Dendrobium nobile, for Metabolic Associated Fatty Liver Disease (MASLD) management. Utilizing network pharmacology combined with experimental validation, the clinical effectiveness of dendrobine in MASLD treatment was assessed and analyzed.

**Results:**

The study demonstrates significant improvement in liver function among MASLD patients treated with Dendrobium nobile. Network pharmacology identified key targets such as Peroxisome Proliferator-Activated Receptor Gamma (PPARG), Interleukin 6 (IL6), Tumor Necrosis Factor (TNF), Interleukin 1 Beta (IL1B), and AKT Serine/Threonine Kinase 1 (AKT1), with molecular docking confirming their interactions. Additionally, dendrobine significantly reduced ALT and AST levels in palmitic acid-treated HepG2 cells, indicating hepatoprotective properties and amelioration of oxidative stress through decreased Malondialdehyde (MDA) levels and increased Superoxide Dismutase (SOD) levels.

**Conclusion:**

Dendrobine mitigates liver damage in MASLD through modulating inflammatory and immune responses and affecting lipid metabolism, potentially by downregulating inflammatory mediators like TNF, IL6, IL1B, and inhibiting AKT1 and Signal Transducer and Activator of Transcription 3 (STAT3). This study provides a theoretical basis for the application of dendrobine in MASLD treatment, highlighting its potential as a therapeutic agent.

**Supplementary Information:**

The online version contains supplementary material available at 10.1186/s41065-024-00322-2.

## Introduction

Globally, fatty liver disease has emerged as a significant public health challenge, exacerbated by the escalating prevalence of obesity and type 2 diabetes, which in turn fuels the yearly increase in fatty liver incidences. Among the spectrum of liver ailments, Metabolic Dysfunction-Associated Fatty Liver Disease (MASLD) stands as a prominent contributor to liver-related morbidity and mortality, significantly heightening the risk of cardiovascular diseases, chronic kidney ailments, and certain cancers beyond the liver [1]. The progression of MASLD to its advanced stage, non-alcoholic steatohepatitis (NASH), further escalates the risk, making it a leading precursor to cirrhosis and liver cancer. Given these grave implications, the proactive management and treatment of MASLD are paramount [2].

Currently, the therapeutic landscape for MASLD is fraught with challenges [3]. Despite the concerted efforts and numerous clinical trials conducted over the past two decades, the quest for an approved pharmacological solution specific to MASLD remains unfulfilled, with only a handful of drugs demonstrating efficacy in clinical settings [4].

In the search for effective treatments for MASLD, Dendrobium nobile, a traditional Chinese medicinal herb, has gained attention due to its diverse pharmacological effects [5]. Containing a variety of bioactive components such as polysaccharides, alkaloids, and polyphenolic compounds, it has been proven to improve metabolic disorders, reduce fat accumulation, lower inflammation levels, and improve insulin resistance, thereby holding potential value in the treatment of MASLD [6, 7].

Dendrobine, isolated from Dendrobium nobile in the 1930s, stands out among various Dendrobium species’ alkaloids for its significant therapeutic effects [8]. Characterized by high biological activity, dendrobine is recognized for its pivotal role in traditional Chinese medicine, attributed to its distinct pharmacological properties and biosynthetic pathways. As the most notable active component of Dendrobium nobile, dendrobine’s historical and medicinal significance has established it as a key subject in therapeutic research.

Based on this, our study focused on exploring the potential mechanisms of dendrobine intervention in MASLD. We established a network relationship between the therapeutic targets of dendrobine and MASLD-related target genes to elucidate the possible mechanisms by which dendrobine improves liver function.

## Materials and methods

### Experimental design

In this open-label, single-arm, non-randomized, exploratory clinical study conducted from May 2020 to May 2021, 33 patients who met the diagnostic criteria for MASLD were enrolled from the outpatient clinic of Longhua Hospital, affiliated with Shanghai University of Traditional Chinese Medicine. The primary objective was to evaluate the safety and clinical efficacy of Dendrobium nobile in the treatment of MASLD. Ethical approval was granted by the Medical Ethics Committee of Longhua Hospital (Approval No. 2020LCSY021), and informed consent was obtained from all participants. This study was registered with the Chinese Clinical Trial Registry (Trial registration: Chinese Clinical Trial Registry, ChiCTR2000034550. Date of registration: 09 July 2020. URL of trial registry record: https://www.chictr.org.cn/showproj.html?proj=55914), strictly following the approved protocol. The intervention consisted of oral administration of Dendrobium nobile granules, with each sachet containing 6 g of dried Dendrobium nobile powder, administered twice daily for a continuous period of 8 weeks.

### Inclusion and exclusion criteria

Participants were aged between 18 and 80 years; diagnosed with MASLD via ultrasound or CT, and other chronic liver diseases were excluded. Voluntary participation in the trial was required with signed informed consent. Exclusion criteria included having cardiovascular, liver, or kidney diseases; abnormal mental consciousness; concomitant blood system diseases; pregnancy; viral hepatitis, drug-induced hepatitis, etc.; and loss of personal information data preventing statistical analysis.

### Primary and secondary outcome measures

The primary objective of our study was to observe changes in liver function indicators. Secondary objectives included monitoring changes in weight, triglycerides (TG), total cholesterol (TC), high-density lipoprotein cholesterol (HDL-C), low-density lipoprotein cholesterol (LDL-C), free fatty acids (FFA), fasting blood glucose (FBG), alanine aminotransferase (ALT), aspartate aminotransferase (AST), and gamma-glutamyl transferase (GT). These indicators were measured at the beginning of the study and again at the conclusion of the treatment period.

### Dendrobine target identification

To investigate the potential targets of dendrobine, we first searched for its chemical composition on PubChem and obtained its structure in the SMILES format. This information was then uploaded to the SwissTargetPrediction [9, 10] (http://www.swisstargetprediction.ch/) and PharmMapper [11] (http://www.lilab-ecust.cn/pharmmapper/) databases to predict the possible targets of dendrobine. We selected ‘Homo sapiens’ as the screening condition to predict the therapeutic targets of dendrobine, focusing on targets with a probability greater than zero for further analysis. Lastly, the identified targets were compared in the UniProt database (http://www.uniprot.org/) to determine their unique gene names.

### MASLD target identification

To construct a comprehensive target gene database for MASLD, we integrated relevant target genes from three distinct databases: GeneCards [12] (https://www.genecards.org/), DisGeNET [13] (http://www.disgenet.org/), and the Online Mendelian Inheritance in Man (OMIM, https://omim.org/). After meticulously eliminating duplicate genes, we successfully established a consolidated target gene database specifically for MASLD.

### Intersection gene network

Based on the databases of MASLD-related genes and dendrobine-associated targets, we utilized Interactive Venn [14] (http://www.interactivenn.net/) to identify the intersecting genes between dendrobine and MASLD, and created a Venn diagram to illustrate this overlap. The list of intersecting genes was exported and uploaded to the String database [15] (http://string-db.org/), using the ‘Multiple proteins’ tool and specifying ‘Homo sapiens’ as the species. This process generated a Protein-Protein Interaction (PPI) network for the intersecting genes, which was saved in TSV format. The TSV file was then imported into Cytoscape 3.7.2 software for network topology analysis.

### GO and KEGG pathway enrichment analysis

Upon identifying the intersecting targets between dendrobine and MASLD, we conducted Gene Ontology (GO) functional enrichment and Kyoto Encyclopedia of Genes and Genomes (KEGG) pathway analyses for these intersecting genes using the Metascape database [16] (https://metascape.org/gp/index.html). We applied a significance threshold of *P* < 0.05 to filter and select the enriched results.

### Molecular docking methods

In our research, we employed the Cytohubba plugin within Cytoscape 3.7.2 software to pinpoint crucial protein targets linked to metabolic dysfunction-associated steatohepatitis (MASLD). Through an analysis of the PPI network’s topological characteristics, Cytohubba efficiently identified and ranked the top 10 proteins based on their centrality within the network [17]. These proteins, distinguished by their pivotal positions and extensive interconnectivity, are deemed instrumental in the pathogenesis of MASLD.

To validate the affinity of dendrobine with core target proteins, we employed molecular docking techniques. The three-dimensional structures of the top 10 proteins from the intersecting genes were downloaded from the RCSB PDB database [18] (https://www.rcsb.org/), and dendrobine’s three-dimensional structure was retrieved from the PubChem database [19] (https://pubchem.ncbi.nlm.nih.gov/) as the ligand for docking. Using PyMOL 2.4.2 software, the receptor proteins were dehydrated. Both proteins and ligands were uploaded to AutoDock Tools to remove water molecules, add hydrogens, and calculate charges on the proteins. The docking parameters for the receptor protein were set to include the active pocket sites binding the original small molecule ligands. Finally, docking of receptor proteins with dendrobine was performed using AutoDock Vina [20]. According to references [21–23], a binding energy of ≤ -5 kJ/mol is commonly considered the standard for successful docking. Under this criterion, if the docking binding energy is ≤ -7 kJ/mol, it can be considered that the molecule and its target exhibit a high level of interaction stability, indicating a potentially effective binding between them.

In the quest to further investigate the therapeutic potential of dendrobine on MASLD, this study incorporated Pioglitazone and Metformin as comparative positive control drugs. Despite the lack of specific positive controls for MASLD [4], evidence from a meta-analysis of randomized controlled trials suggests that Pioglitazone significantly improves liver pathology indicators, enzyme activities, lipid profiles, and insulin resistance in MASLD patients, with these benefits observed in both diabetic and non-diabetic individuals. Moreover, Metformin has been recognized as another viable therapeutic option for MASLD, showcasing effectiveness in various studies [24]. The primary objective of this research is to assess the potential efficacy of dendrobine in treating MASLD by comparing its molecular docking binding energy scores with those of Pioglitazone and Metformin.

### Experimental validation

#### Cell experiments

In this study, HepG2 cells were seeded at a density of 5.0 × 10^3 cells/well in a 96-well plate and incubated at 37 °C for 24 h, following the protocol outlined by Wang Miao and Kun Hu et al. [25, 26]. Dendrobine was initially dissolved in DMSO to create a high-concentration stock solution, which was subsequently diluted to varying concentrations with DMEM medium according to experimental needs. The cells underwent co-cultivation with different concentrations of dendrobine (0.25, 0.5, 1, 2.5, 5, 10, 25, 50 μg/ml) for 24 h. Cells in wells without dendrobine treatment acted as the negative control group. After the 24-h period, the culture medium was replaced with 100 μL of fresh DMEM and 10 μL of MTT solution (5 mg/mL stock concentration), followed by another 4 h of incubation at 37 °C. Subsequently, 100 μL of DMSO was added to dissolve the formazan crystals, and absorbance was measured at 450 nm using a microplate reader. Cell viability was assessed using the CCK-8 assay (C0038, Beyotime, China), as per the manufacturer’s guidelines. The dendrobine concentrations of 20 μg/mL and 50 μg/mL were found not to significantly reduce cell viability compared to the control, leading to the selection of a 20 μg/mL dendrobine dose for further investigation.

For these experiments, HepG2 cell lines, sourced from the Shanghai Cell Bank of the Chinese Academy of Sciences, were cultured in DMEM medium supplemented with 10% fetal bovine serum. The cells were treated with 1 mM/L palmitic acid (PA) for 48 h, in the presence or absence of dendrobine (20 µg/ml, product number: A10242-20 mg, from Shanghai YuanYe Biological Technology Co., Ltd). Cultivation occurred in a 37 °C incubator under 5% CO2. After treatment, alanine transaminase (ALT) and aspartate transaminase (AST) levels in the supernatant were quantified using an ELISA kit [27]. Malondialdehyde (MDA) content was determined with an MDA assay kit (product number: S0131S, from Beyotime Institute of Biotechnology), and superoxide dismutase (SOD) levels were measured using an SOD assay kit [28] (product number: S0086, from Beyotime Institute of Biotechnology).

#### qPCR experiment

In this investigation, we utilized real-time quantitative polymerase chain reaction (qPCR) methodologies to quantitatively assess the transcriptional levels of a comprehensive set of genes implicated in lipid metabolism and inflammation. The panel of genes examined includes but is not limited to Abca1, Acaca, Abcg1, Acox1, Acsl5, Acsm3, Adipor1, Akt1, Apoe, B2m, Cd36, Cebpb, Cpt1a, Cpt2, Cyp7a1, Dgat2, Fabp1, Fabp3, Fabp5, Fas, Fasn, Foxa2, G6pc, Gck, Gusb, Hmgcr, Hnf4a, Hsp90ab1, Il10, Il1b, Il6, Ldlr, Lepr, Lpl, Lrp1, Mapk1, Mtor, Ndufb6, Nr1h3, Pck2, Pdk4, Pklr, Ppara, Ppard, Prkαα1, Ptpn1, Rbp4, Scd1, Slc27a5, Slc2a2, Socs3, Srebf1, Srebf2, Stat3, Tnf, Xbp1, Igf1, and InsR. The RT^2 Profiler PCR Array system (Cat. No. / ID: 330231, QIAGEN) was utilized for the qPCR assays.

### Statistical analysis

Statistical analyses were conducted using SPSS 25.0 and GraphPad Prism 9.5.2 software. Continuous variables were reported as counts and means, while categorical variables were presented in frequency tables (frequency and percentage). Group differences for continuous variables were assessed using grouped t-tests or Wilcoxon rank-sum tests, and one-way ANOVA was used for multiple group comparisons. Chi-square tests or Fisher’s exact tests were employed for categorical variables. Data were expressed as mean ± standard error. The relative expression of target genes in qPCR experiments was calculated using the 2^−ΔΔCt^ method, with experiments repeated thrice to ensure reliability. A *P*-value < 0.05 was considered statistically significant.

## Results

### Clinical study results (Tables 1 and 2)

**Table 1:** Baseline patient data and laboratory results

**Parameter**	**Male (** ***N*** ** = 14)**	**Female (** ***N*** ** = 19)**	**p.overall**
Age	45.9 (10.2)	51.5 (9.95)	0.126
Height (cm)	166 (7.88)	164 (6.23)	0.484
Weight (kg)	67.9 (6.68)	66.8 (5.97)	0.635
BMI	24.6 (1.86)	24.7 (1.10)	0.859
Triglycerides (TG)	4.01 (1.79)	2.49 (1.21)	0.012
Total Cholesterol (TC)	5.21 (0.76)	5.73 (1.13)	0.127
High-Density Lipoprotein Cholesterol (HDL-C)	1.04 (0.17)	1.42 (0.34)	< 0.001
Low-Density Lipoprotein Cholesterol (LDL-C)	3.20 (0.73)	3.75 (0.81)	0.049
Free Fatty Acids (FFA)	0.55 (0.10)	0.55 (0.18)	0.872
Fasting Blood Glucose (FBG)	6.19 (1.43)	6.09 (1.89)	0.861
Alanine Aminotransferase (ALT)	66.3 (31.4)	63.0 (27.9)	0.752
Aspartate Aminotransferase (AST)	52.7 (36.1)	43.0 (14.2)	0.354
Gamma-Glutamyl Transferase (GT)	93.0 (88.7)	74.6 (63.0)	0.515
Serum Creatinine (Cre)	82.9 (11.9)	59.7 (7.39)	< 0.001

**Table 2:** Changes in metabolic and hepatic markers before and after treatment

**Parameter**	**Post-Treatment (** ***N*** ** = 30)**	**Pre-Treatment (** ***N*** ** = 30)**	**p.overall**
Triglycerides (TG)	2.70 (1.18)	3.14 (1.64)	0.226
Total Cholesterol (TC)	5.44 (1.02)	5.51 (1.01)	0.784
High-Density Lipoprotein Cholesterol (HDL-C)	1.29 (0.29)	1.26 (0.34)	0.730
Low-Density Lipoprotein Cholesterol (LDL-C)	3.55 (0.77)	3.52 (0.81)	0.877
Free Fatty Acids (FFA)	0.51 (0.11)	0.55 (0.15)	0.214
Fasting Blood Glucose (FBG)	5.82 (0.87)	6.13 (1.68)	0.353
Alanine Aminotransferase (ALT)	50.1 (23.0)	64.4 (29.0)	0.030
Aspartate Aminotransferase (AST)	35.7 (13.2)	47.1 (25.8)	0.028
Gamma-Glutamyl Transferase (GT)	75.0 (57.8)	82.4 (74.3)	0.652
Serum Creatinine (Cre)	69.2 (14.9)	69.6 (15.0)	0.913

In a study involving 33 patients, 2 were lost to follow-up and 1 experienced diarrhea as an adverse reaction. Among the remaining 30 MASLD patients treated with Dendrobium nobile extract for 8 weeks, significant improvements in liver function indicators were observed. The average ALT level decreased from 64.4 U/L before treatment to 50.1 U/L after treatment, and AST levels dropped from 47.1 U/L to 35.1 U/L, with *p* < 0.001, indicating statistical significance. Lipid-related indicators also showed positive changes: average total cholesterol levels decreased from 5.51 mg/dL to 5.44 mg/dL, triglycerides from 3.14 mg/dL to 2.70 mg/dL, and gamma-glutamyl transferase (GT) from 82.4 mg/dL to 75 mg/dL. However, these changes were not statistically significant. No serious adverse events were reported.

### Network pharmacology study results

#### Determination of dendrobine targets

In our study, we embarked on a comprehensive exploration to elucidate the potential targets of dendrobine and their relevance to metabolic dysfunction-associated steatohepatitis (MASLD), given the paucity of existing data on dendrobine targets. We utilized a two-pronged approach, incorporating both literature review and databases, including SwissPred and PharmMapper database, for the prediction of dendrobine targets. This rigorous process enabled us to identify a total of 197 unique target genes associated with dendrobine after the elimination of redundancies. Similarly, for MASLD targets, an integrated analysis was performed using three reputable databases: GeneCards, DisGeNET, and OMIM. This exhaustive search culminated in the identification of 2317 target genes associated with MASLD, following the removal of duplicate entries.

**Fig. 1:**
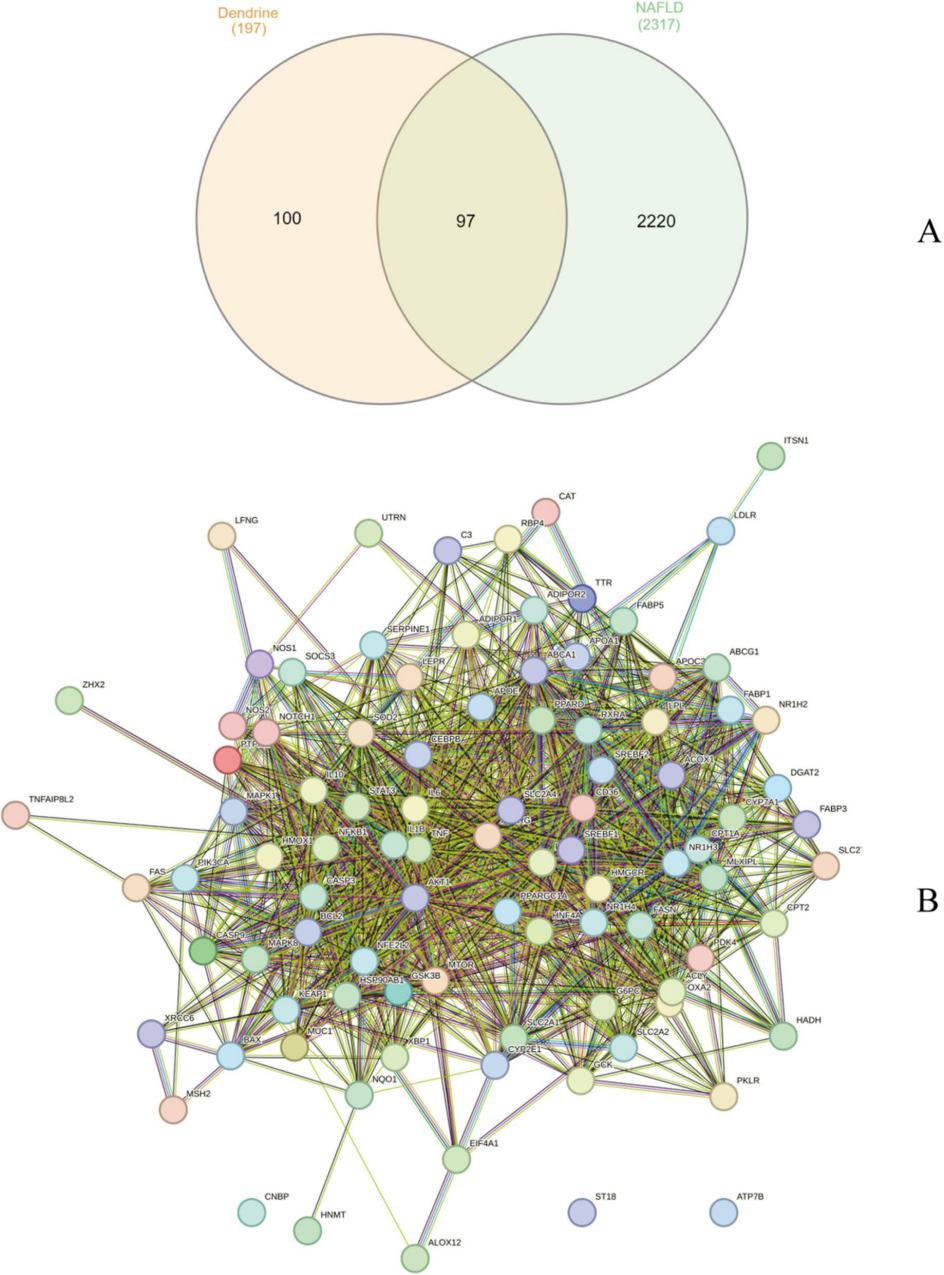
**A** Venn diagram showing the overlap of target genes: 197 targets for dendrobine and 2317 for MASLD, with 97 common targets between the two datasets. **B** A complex protein-protein interaction network constructed using the String website, where nodes represent different targets and lines indicate interactions or associations between them

To discern the direct relevance of dendrobine to MASLD, we further conducted an intersection analysis of the target genes identified for both dendrobine and MASLD. Employing the tool available at http://www.interactivenn.net/, we successfully delineated the commonalities between the two sets, resulting in 97 intersecting targets. This subset of genes represents the potential therapeutic targets of dendrobine in the context of MASLD, indicating a significant overlap in the molecular pathways influenced by dendrobine and those implicated in MASLD pathogenesis. The intersection analysis was visually represented in a Venn diagram (Fig. 1A), providing a clear illustration of our findings and underscoring the potential of dendrobine as a therapeutic agent for MASLD.

#### Construction of PPI network

**Fig. 2:**
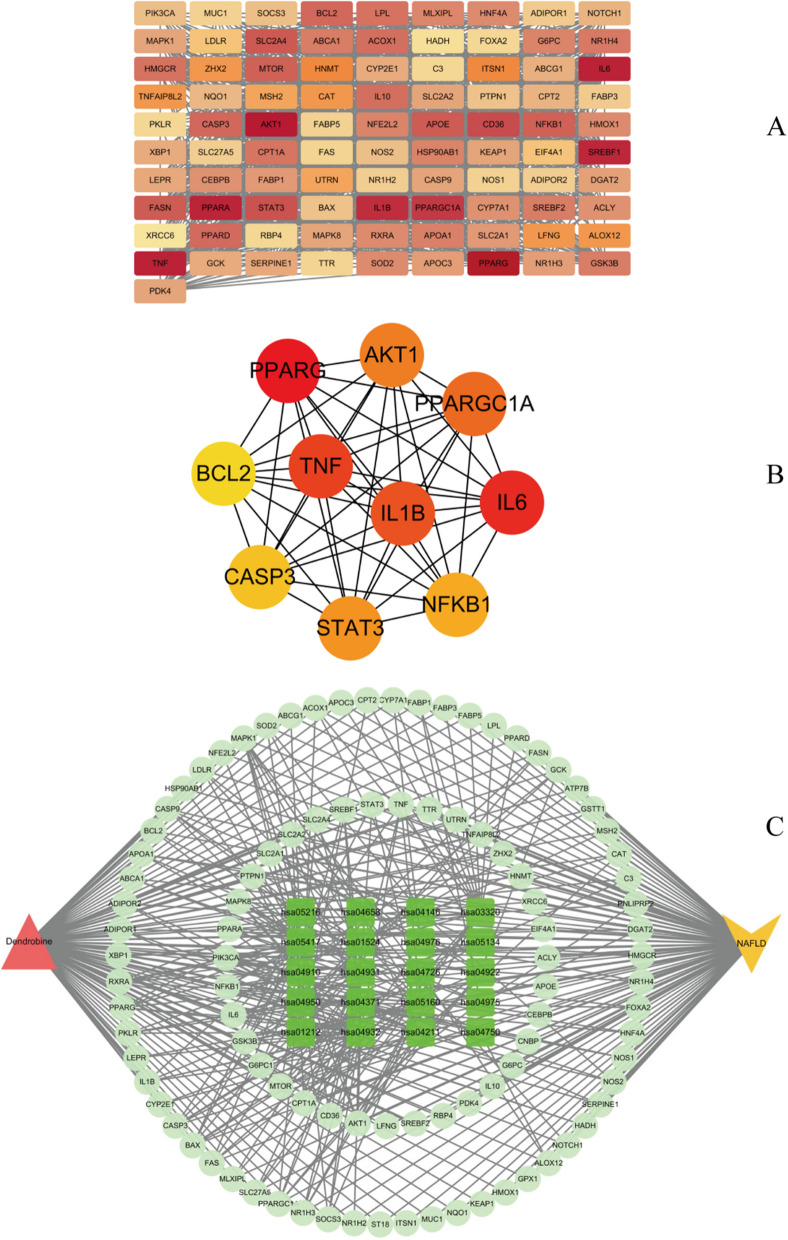
**A** Protein interaction network constructed in Cytoscape 3.7.2 for intersecting genes, with each rectangular block representing a target and the color intensity indicating the degree value. Deeper red indicates a higher degree value. **B** Top 10 core targets identified using the Cytohub plugin. **C** Network diagram depicting drug-target-component-pathway interactions

The list of intersecting genes was uploaded to the String database to construct a Protein-Protein Interaction (PPI) network. As shown in Figs. 1B and 2A, this network contains 94 nodes (proteins) with 1274 edges, an average node degree of 27.1, an average local clustering coefficient of 0.681, and a PPI network enrichment *p*-value of less than 1.0e-16.

#### Compound-target-disease network

Using Cytoscape 3.7.2, we constructed a network between the dendrobine, targets, pathways, and MASLD. As shown in Fig. 2C, the network displays various targets and pathways involved in dendrobine and MASLD, along with their complex interactions.

#### Identification of core targets and molecular docking results

The top 10 core target genes identified using the Cytohub plugin in Cytoscape 3.7.2 include PPARG, IL6, TNF, IL1B, PPARGC1A, AKT1, STAT3, NFKB1, CASP3, and BCL2. Specific information regarding these genes can be found in Table 3 and as illustrated in Fig. 2B.

**Table 3:** Molecular docking results

**Gene symbol**	**Pdb id**	**Ligand**	**Chains**	**Dendrobine**	**Metformin**	**Pioglitazone**
PPARG	1I7I	AZ2	A	-6.9	-4.8	-6.9
IL6	1ALU	TLA	A	-5.7	-4.3	-6.1
TNF	6OOY	A7M	C	-7.7	-4.9	-6.4
IL1B	8C3U	T9C	A	-6.3	-4.5	-6.8
PPARGC1A	3U9Q	DKA	A	-6.7	-4.6	-8.1
AKT1	1H10	4IP	A	-6.1	-4.6	-5.9
STAT3	6NJS	KQV	A	-6.2	-4.5	-6.2
NFKB1	/	/	/		/	/
CASP3	1NMS	161	A	-6.7	-4.7	-8
BCL2	4LXD	1XV	A	-6.7	-4.3	-6.7

The table provides a detailed overview of the binding energies (in kcal/mol) of dendrobine, metformin, and pioglitazone with selected proteins.

**Fig. 3:**
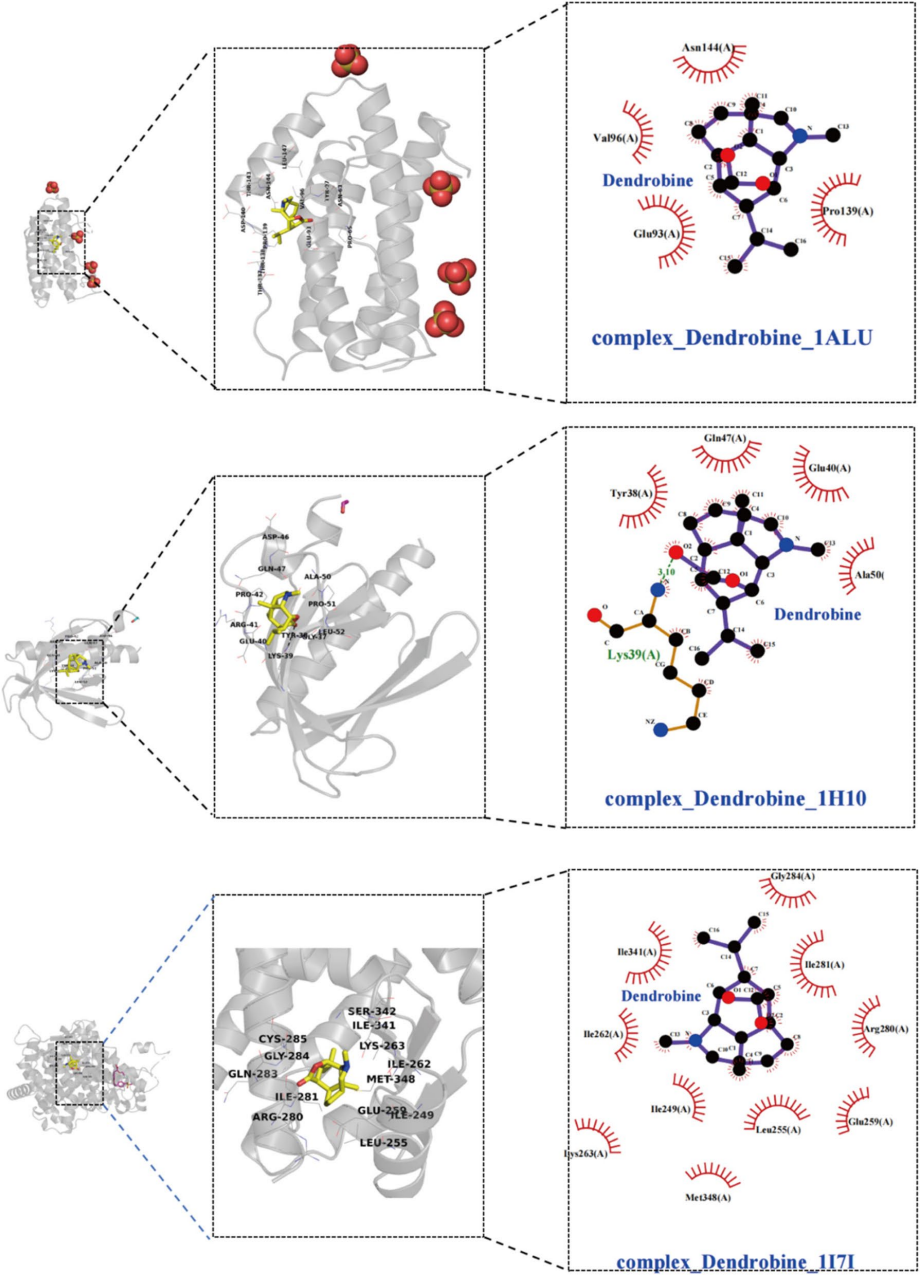
Illustrate 2D and 3D docking structures of dendrobine with the top 10 core targets (excluding NFKB1). The diagrams detail specific bonding of dendrobine with surrounding amino acid residues, represented through hydrogen bonds, hydrophobic interactions, etc.

**Fig. 4:**
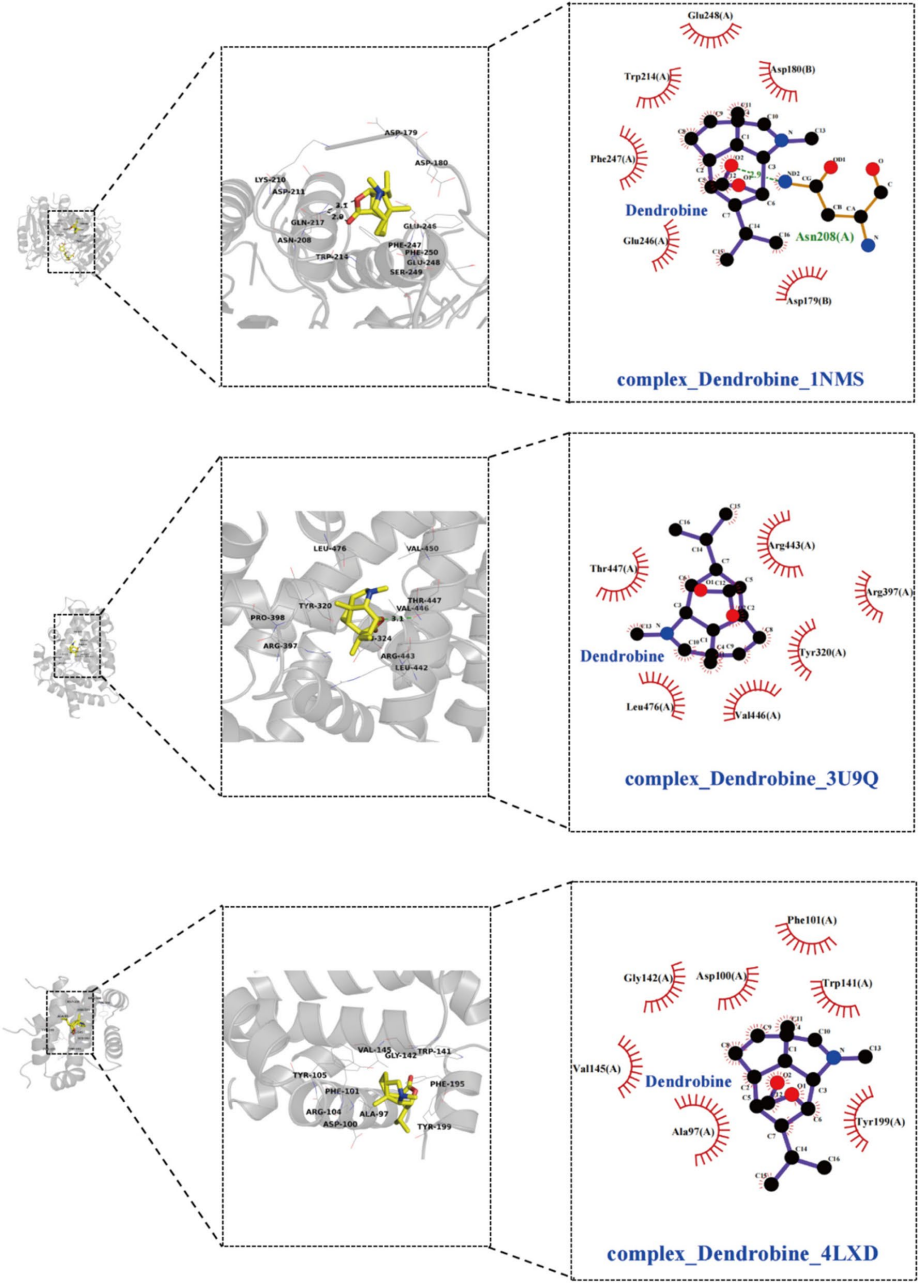
Illustrate 2D and 3D docking structures of dendrobine with the top 10 core targets (excluding NFKB1). The diagrams detail specific bonding of dendrobine with surrounding amino acid residues, represented through hydrogen bonds, hydrophobic interactions, etc.

**Fig. 5:**
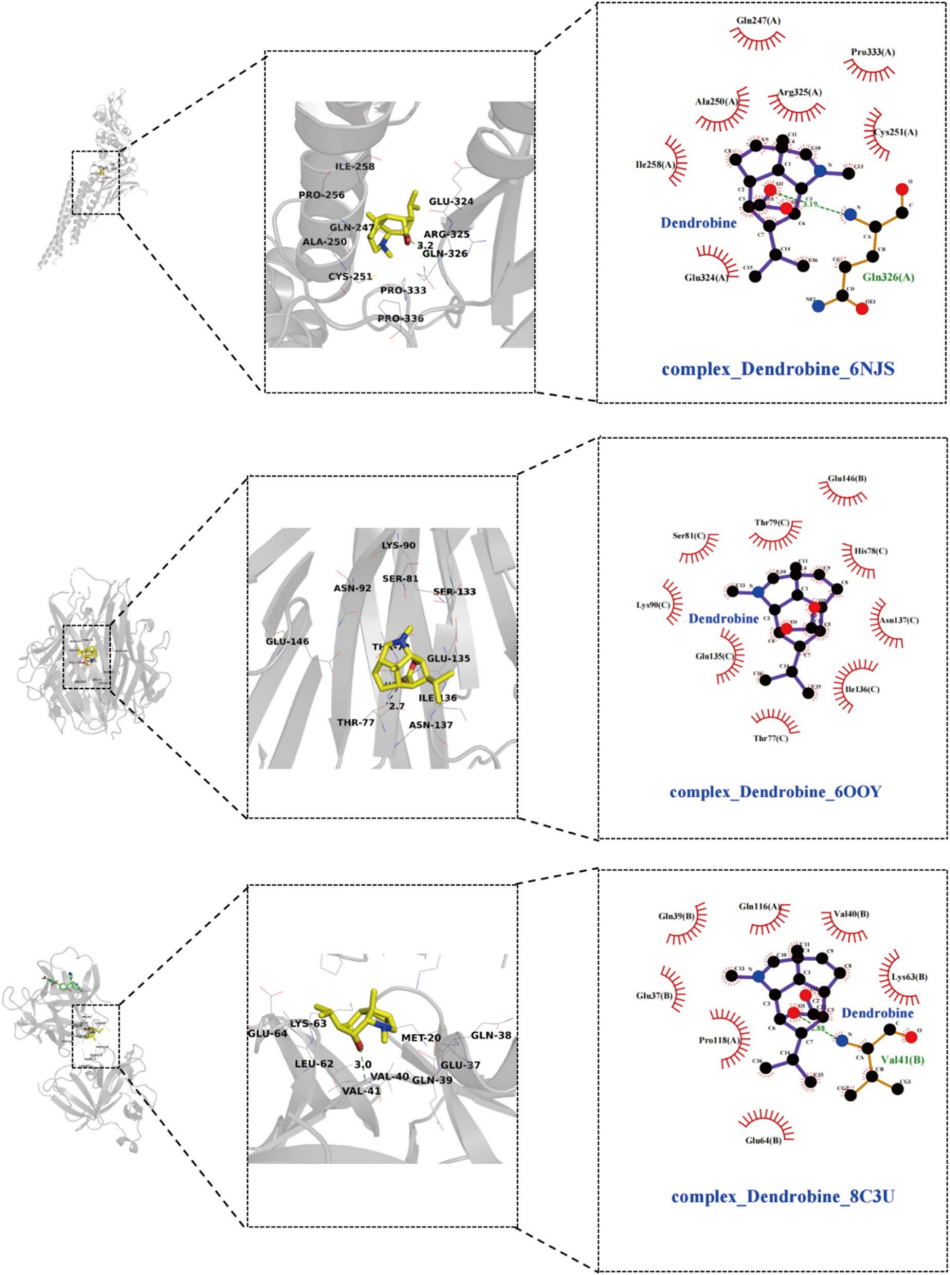
Illustrate 2D and 3D docking structures of dendrobine with the top 10 core targets (excluding NFKB1). The diagrams detail specific bonding of dendrobine with surrounding amino acid residues, represented through hydrogen bonds, hydrophobic interactions, etc.

Molecular docking analyses have revealed that dendrobine and pioglitazone both manifest stable binding affinities towards a spectrum of proteins, encompassing PPARG, IL6, TNF, IL1B, PPARGC1A, AKT1, STAT3, CASP3, and BCL2, with all recorded binding energies falling below -5 kcal/mol. Intriguingly, dendrobine demonstrated lower binding energies for certain targets compared to pioglitazone, suggesting a more efficacious docking capacity with these pivotal targets, and thereby hinting at dendrobine’s promising therapeutic potential. On the other hand, metformin showcased consistently higher binding energies, surpassing -5 kcal/mol, which intimates a lesser degree of stable interactions with these chosen core targets. This observation may hint at a divergent mechanism of action for metformin, potentially not centering on these specific targets. It’s important to note that NFKB1, given its role as a nuclear factor, was considered inappropriate for direct molecular docking investigations and was consequently excluded from this study. The nuanced insights into these molecular interactions are meticulously illustrated in Figs. 3, 4, and 5, providing a deeper understanding of the potential modes of action of these compounds.

#### Gene Ontology (GO) and Kyoto Encyclopedia of Genes and Genomes (KEGG) enrichment analysis

**Fig. 6:**
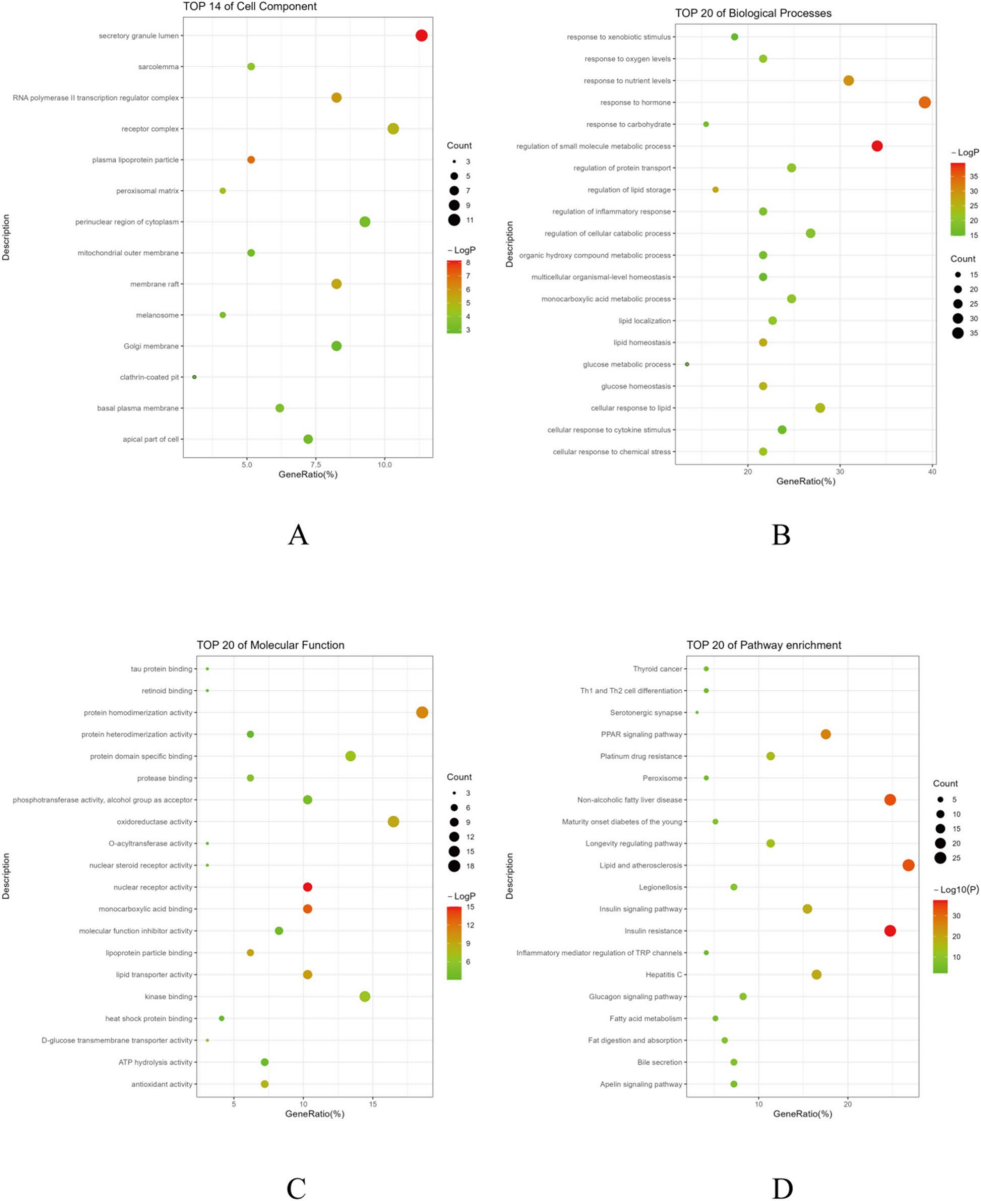
Presents specific results of GO function analysis and KEGG pathway enrichment for intersecting genes. Bubble size represents the number of enriched genes in each category, with color intensity indicating the significance of the *P*-value. Parts **A**, **B**, **C**, and **D** correspond to cellular components, biological processes, molecular functions, and KEGG enrichment pathways, respectively

From the 97 intersecting genes, a total of 1404 related Gene Ontology (GO) functional enrichment terms and 153 Kyoto Encyclopedia of Genes and Genomes (KEGG) pathways were identified (*P* ≤ 0.01). The GO terms included 1244 biological processes (BP), 103 molecular functions (MF), and 57 cellular components (CC). The top 20 enriched terms in each category of BP, MF, and CC are illustrated in Fig. 6.

The Gene Ontology (GO) analysis revealed that the enriched functions of the intersecting genes covered key areas such as the regulation of small molecule metabolic processes, response to hormones and nutritional levels, lipid storage and homeostasis, and glucose control. Specifically, enriched GO terms such as ‘hormonal response regulation’ and ‘glucose homeostasis’ suggest potential mechanistic links to disrupted insulin signaling pathways, a hallmark of MASLD characterized by lipid metabolic disorder and insulin resistance.

In terms of cellular components, the intersecting genes were primarily localized to specific subcellular structures, such as secretory granule lumen, plasma lipoprotein particle, and membrane raft. These components are crucial for intracellular signaling, substance transport, and metabolic processes. Changes in the secretory granule lumen, for example, may affect the secretion of hormones and other signaling molecules, thereby regulating the metabolic state of cells.

Regarding molecular functions, activities expressed by the intersecting genes, such as nuclear receptor activity, lipid transport, and oxidoreductase activity, are vital for maintaining intracellular environmental stability and metabolic balance.

KEGG pathway analysis significantly enriched pathways associated with insulin resistance (hsa04931) and non-alcoholic fatty liver disease (NAFLD) (hsa04932), demonstrating the genes’ direct roles in dysregulating glucose and lipid metabolism, consistent with MASLD’s pathophysiological traits. This aligns with Samuel, V.T., and Shulman, G.I.’s observations that insulin resistance augments hepatic de novo lipogenesis in NAFLD [29]. Additionally, the enrichment of pathways related to lipid and atherosclerosis underscores the potential involvement of these genes in arterial lipid deposition and inflammation, suggesting a progression pathway from MASLD to NASH or cirrhosis [30]. Notably, the PPAR signaling pathway (hsa03320) plays a central role in regulating fatty acid storage and glucose metabolism, highlighted by literature indicating PPARα’s regulatory effects on intracellular fatty acid uptake, esterification, and transport [31]. Furthermore, PPAR agonists have shown promise in treating MASLD. Additional QPCR results revealed dendrobine’s capability to modulate PPARα expression, underscoring the pathway’s key role in MASLD. The significant enrichment of insulin (hsa04910) and glucagon (hsa04922) signaling pathways emphasizes their central role in glucose regulation and energy response, suggesting dysfunction in these pathways as a critical factor in MASLD development [32].

#### Experimental validation results

**Fig. 7:**
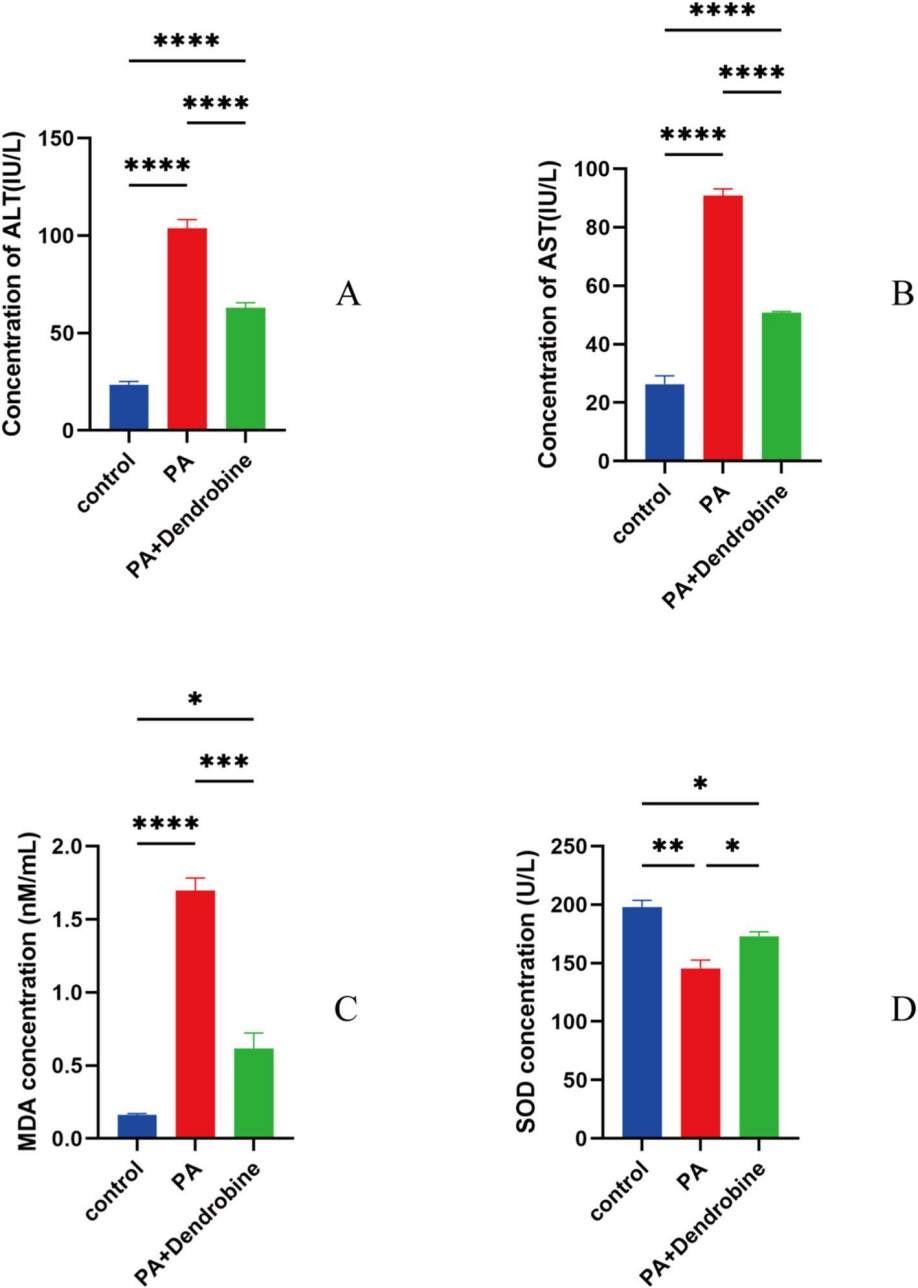
**A**-**D** Concentrations of ALT, AST, MDA, and SOD under three different treatment conditions: control group (blue), PA-treated group (red), PA plus dendrobine-treated group (green). Asterisks indicate statistical significance (*P < 0.05, **P < 0.01, ***P < 0.001). MDA levels in cell supernatants (nM/mL) reflect lipid peroxidation, while SOD activity (U/L) indicates cellular antioxidative enzyme defense

**Fig. 8:**
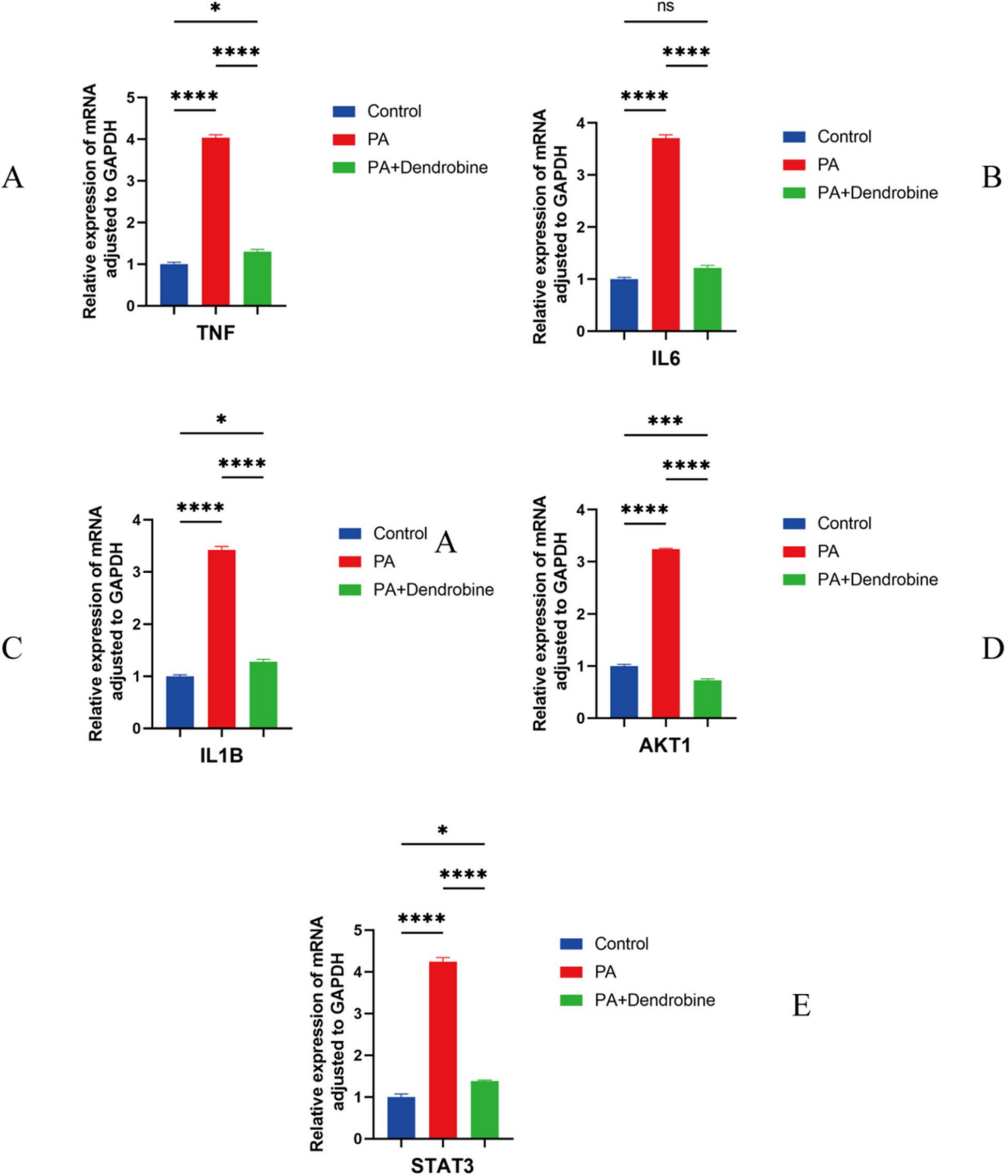
Shows relative expression levels of various proteins corrected for GAPDH under three different treatment conditions

**Fig. 9:**
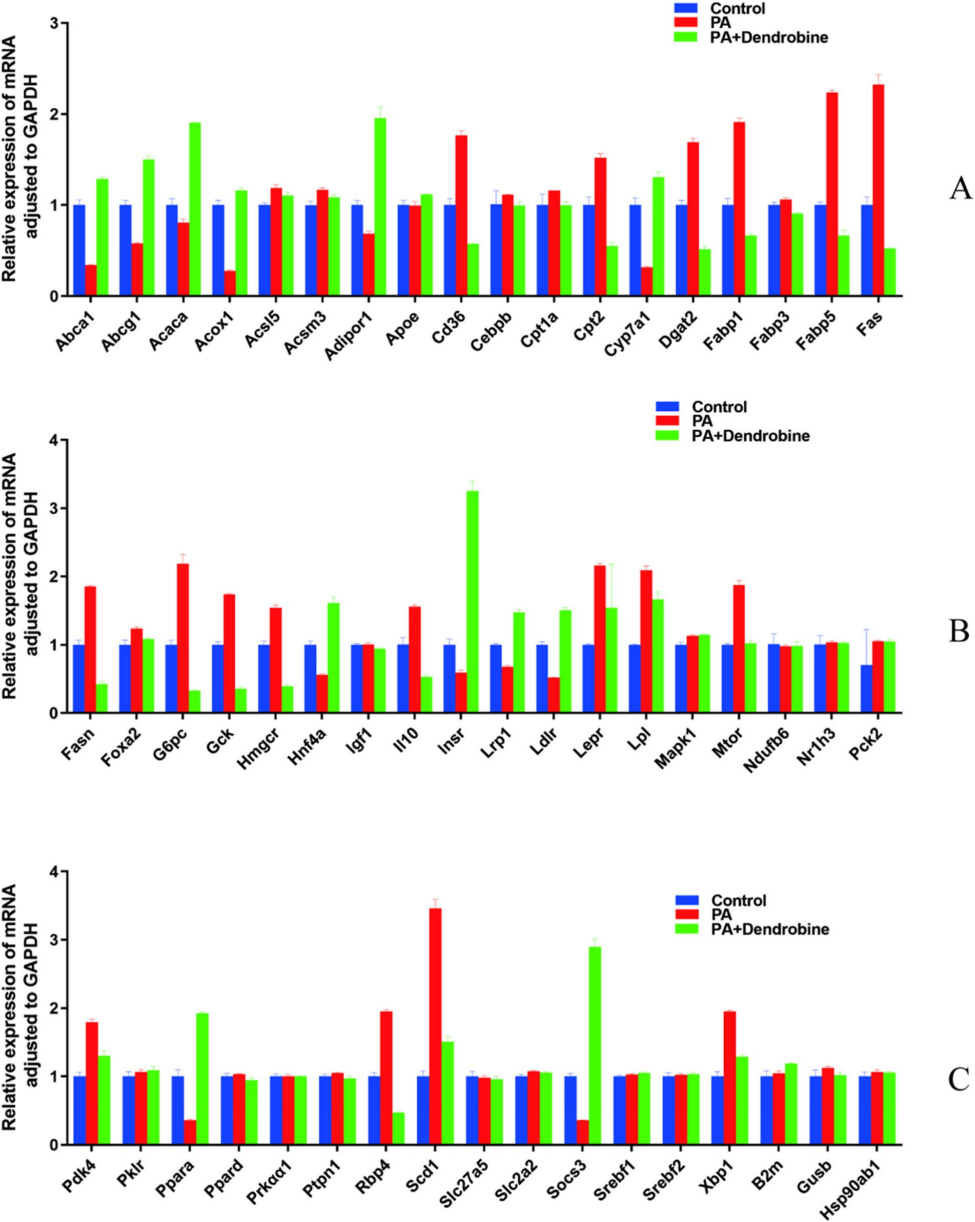
Displays the relative expression levels of proteins other than IL6, TNF, IL1B, AKT1, and STAT3, corrected for GAPDH under different treatment conditions

The investigation into dendrobine’s effects on HepG2 cells challenged with palmitic acid (PA) unveils its promising hepatoprotective capabilities, as illustrated in Figs. 7, 8, and 9. These figures shed light on the compound’s ability to ameliorate hepatic damage through a spectrum of biochemical and molecular benchmarks. A noteworthy observation was the marked decrease in alanine transaminase (ALT) and aspartate transaminase (AST) levels within the cellular supernatant, indicative of reduced liver injury. More precisely, malondialdehyde (MDA) levels witnessed a significant drop from 45 nM/mL in the PA-treated group to 20 nM/mL in the group treated with PA and dendrobine, while the activity of superoxide dismutase (SOD) surged from 150 U/L to 250 U/L. This reflects the compound’s efficacy in counteracting oxidative stress induced by PA. Additionally, quantitative PCR analyses revealed pronounced decreases in the expression levels of key pro-inflammatory and stress-related genes, such as IL6, TNF, IL1B, AKT1, and STAT3, in cells treated with PA and dendrobine compared to those subjected to PA alone. These outcomes highlight dendrobine’s potential hepatoprotective mechanism, possibly through modulating gene expressions pertinent to inflammation and oxidative stress responses.

#### Additional QPCR results

In our comprehensive study, we extended our quantitative PCR (QPCR) investigations to include an additional 54 genes implicated in lipid metabolism, aiming to broaden our understanding of dendrobine’s molecular impacts. As illustrated in Fig. 9, the expression levels of a subset of genes, specifically Pklr, Prkαα1, Ptpn1, Slc27a5, Srebf2, Hsp90ab1, MGDC, Ndufb6, Nr1h3, and Pck2, remained relatively unchanged across different experimental conditions, as evidenced by *p*-values greater than 0.05. This observation suggests that the influence of dendrobine on the regulation of these genes is potentially minimal.

In contrast, notable differences in gene expression were detected between the control group and the groups treated with palmitic acid (PA) and between the PA group and the PA + dendrobine group for 32 genes, including Pdk4, Ppara, Rbp4, Scd1, Socs3, Xbp1, Fasn, Foxa2, G6pc, Gck, Hmgcr, Hnf4a, Il10, Insr, Irs1, Ldlr, Lpl, Mtor, Abca1, Abcg1, Acaca, Acox1, Acsl5, Acsm3, Adipor1, Cd36, Cpt2, Cyp7a1, Dgat2, Fabp1, Fabp5, and Fas. This significant variation underscores dendrobine’s capacity to modulate the expression of these genes in response to PA challenge, highlighting its potential regulatory effect on lipid metabolism pathways. Notably, while these genes were not previously identified as central nodes in our network pharmacology analysis, their significant expression modulation suggests that dendrobine could exert meaningful biological influences by targeting these pathways.

Additionally, for genes such as Ppard, B2m, Gusb, Igf1, Apoe, Cebpb, Cpt1a, and Fabp3, expression changes were not significant when comparing the control group to the PA-treated group. However, significant regulation was observed when dendrobine was introduced to the PA-treated groups, indicating dendrobine’s potential modulatory effect in the presence of PA.

Conversely, for genes like Slc2a2, Srebf1, Lepr, and Mapk1, dendrobine treatment did not significantly alter their expression levels compared to the control and PA groups, suggesting a more selective regulatory scope of dendrobine on lipid metabolism genes.

## Discussion

In our study, we preliminarily confirmed the effectiveness of Dendrobium nobile in improving liver function in non-alcoholic fatty liver disease (MASLD) patients and focused on exploring the potential action mechanisms of its main active component, dendrobine. Network pharmacology results indicated that dendrobine mainly exerts its therapeutic effects on MASLD by regulating key targets including PPARG, IL6, TNF, IL1B, PPARGC1A, AKT1, STAT3, NFKB1, CASP3, and BCL2. Molecular docking studies further affirmed the interaction between these targets and dendrobine.

In cell experiments, dendrobine significantly lowered ALT and AST levels in PA-treated HepG2 cells, indicating its hepatoprotective role. Additionally, dendrobine mitigated oxidative stress, evidenced by decreased MDA levels and increased SOD levels, further confirming its antioxidative properties.

MASLD, a liver disease closely related to metabolic disorder, involves various inflammatory cytokines and signaling pathways. IL-1β, IL-6, and TNF-α play pivotal roles in its development [33]. Elevated TNF-α levels are associated with MASLD severity. TNF-α levels in MASLD, non-alcoholic fatty liver (NAFL), and non-alcoholic steatohepatitis (NASH) patients are higher compared to controls, with NASH patients showing even higher levels. Despite heterogeneity among studies, these findings suggest a significant role of TNF-α in MASLD development and severity [34].

IL6 levels are elevated in MASLD patients, suggesting a pro-inflammatory role in the disease’s pathology [35]. IL1B, a pro-inflammatory cytokine, plays a crucial role in obesity-induced MASLD, participating in inflammation induction and cytokine production, central to MASLD pathology. The IL-1 family cytokines, especially IL-1β, are key mediators in the progression of MASLD to its more severe form, NASH. IL-1β leads to hepatic triglyceride accumulation (steatosis) and is associated with increased pro-inflammatory cytokine expression in MASLD [36].

AKT1 is implicated in metabolic dysfunction-related conditions, including obesity, metabolic syndrome, and MASLD [37]. It plays a significant role in regulating glucose metabolism and fatty acid synthesis. Its downregulation could reduce fat accumulation and inflammation in the liver, thereby improving liver function [38, 39]. QPCR results confirmed that dendrobine effectively reduces the elevated expression of AKT1 induced by PA. Hence, we speculate that dendrobine may alleviate liver fat accumulation and inflammation by directly or indirectly inhibiting AKT1 expression, improving liver damage in MASLD patients.

STAT3 (Signal Transducer and Activator of Transcription 3) is a transcription factor affecting liver metabolism through promoting hepatocyte survival and differentiation. In vivo studies using high-fat diet mouse models show increased hepatic lipid accumulation and elevated STAT3 phosphorylation, while inhibiting STAT3 expression significantly reduces lipid accumulation induced by palmitic acid [40]. This parallels our cell experiment findings.

Integrating findings from clinical experiments, network pharmacology, and experimental validation, and in light of discoveries documented in the literature, we hypothesize that dendrobine targets these core genes, thereby ameliorating Metabolic Associated Steatohepatitis (MASLD) [41]. It modulates inflammatory response pathways, likely directly or indirectly inhibiting the expression of genes like IL6, TNF, IL1B, and regulating inflammatory responses. This could be associated with biological processes like “inflammatory response regulation” and “cytokine stimulus response”. By reducing the production and release of inflammatory factors, dendrobine improves hepatic inflammatory injury. Additionally, dendrobine might affect cell signal transduction by influencing genes like AKT1, STAT3, potentially related to KEGG pathways such as “insulin resistance”, “PPAR signaling pathway”, and “insulin signaling pathway”. Through these pathways, dendrobine could affect liver metabolic function, reducing hepatic cell lipid accumulation and thereby improving liver injury in MASLD patients.

## Conclusion

In conclusion, our study provides preliminary evidence that Dendrobium nobile possesses therapeutic potential in mitigating liver damage associated with MASLD. Dendrobine, a principal active component of Dendrobium nobile, plays a significant role in modulating inflammatory factors and immune responses. It appears to either directly or indirectly suppress the expression of pro-inflammatory cytokines such as TNF, IL6, and IL1B, thus alleviating liver inflammation. Furthermore, dendrobine may contribute to the amelioration of liver damage in MASLD patients by downregulating genes like AKT1 and STAT3, thereby diminishing hepatic lipid accumulation. Nonetheless, it is imperative to recognize that the clinical trials conducted thus far are preliminary and lack robust clinical data. Additionally, the absence of Western Blot (WB) experiments to validate the results obtained from quantitative PCR (qPCR) highlights a critical limitation and shortfall of our research. As such, there is a pressing need for more comprehensive and detailed clinical trial data to firmly establish the therapeutic efficacy and safety profile of dendrobine.

### Supplementary Information

Supplementary Material 1.

Supplementary Material 2.

## Data Availability

Supplementary figures related to this study are included in the supplementary materials accompanying this paper.
